# The role of PLCγ2 in immunological disorders, cancer, and neurodegeneration

**DOI:** 10.1016/j.jbc.2021.100905

**Published:** 2021-06-19

**Authors:** Jacob T. Jackson, Elisabeth Mulazzani, Stephen L. Nutt, Seth L. Masters

**Affiliations:** 1 Immunology Division, The Walter and Eliza Hall Institute of Medical Research, Parkville, Victoria, Australia; 2 Inflammation Division, The Walter and Eliza Hall Institute of Medical Research, Parkville, Victoria, Australia; 3 Department of Medical Biology, University of Melbourne, Parkville, Victoria, Australia; 4 Immunology Laboratory, Guangzhou Institute of Paediatrics, Guangzhou Women and Children’s Medical Centre, Guangzhou, Guangdong, China

**Keywords:** phospholipase C, cancer, neurodegeneration, immunodeficiency, inflammation, Aβ, amyloid β, AD, Alzheimer’s disease, APLAID, PLCγ2-associated antibody deficiency and immune dysregulation with autoinflammation, BCR, B cell receptor, BM, bone marrow, Cbl, c-Casitas B-lineage lymphoma, CLL, chronic lymphocytic leukemia, cSH2, C-terminal Src homology 2, CVID, common variable immunodeficiency, DAG, diacylglycerol, DCs, dendritic cells, DLBCL, diffuse large B cell lymphoma, EBL, endemic Burkitt lymphoma, EBV, Epstein–Barr virus, Ig, immunoglobulin, IP_3_, inositol 1,4,5-trisphosphate, PIP_2_, phosphatidylinositol-4,5-bisphosphate, PLAID, PLCγ2-associated antibody deficiency and immune dysregulation, PLCγ2, phosphatidylinositol-specific phospholipase Cγ2, RA, rheumatoid arthritis, RANKL, receptor activator of nuclear factor kappa-B ligand, ROS, reactive oxygen species, SLE, systemic lupus erythematosus, SLP-76, SH2 domain–containing leukocyte protein of 76 kDa, TB, tuberculosis, TCR, T cell receptor

## Abstract

Phosphatidylinositol-specific phospholipase Cγ2 (PLCγ2) is a critical signaling molecule activated downstream from a variety of cell surface receptors that contain an intracellular immunoreceptor tyrosine-based activation motif. These receptors recruit kinases such as Syk, BTK, and BLNK to phosphorylate and activate PLCγ2, which then generates 1D-myo-inositol 1,4,5-trisphosphate and diacylglycerol. These well-known second messengers are required for diverse membrane functionality including cellular proliferation, endocytosis, and calcium flux. As a result, PLCγ2 dysfunction is associated with a variety of diseases including cancer, neurodegeneration, and immune disorders. The diverse pathologies associated with PLCγ2 are exemplified by distinct genetic variants. Inherited mutations at this locus cause PLCγ2-associated antibody deficiency and immune dysregulation, in some cases with autoinflammation. Acquired mutations at this locus, which often arise as a result of BTK inhibition to treat chronic lymphocytic leukemia, result in constitutive downstream signaling and lymphocyte proliferation. Finally, a third group of PLCγ2 variants actually has a protective effect in a variety of neurodegenerative disorders, presumably by increased uptake and degradation of deleterious neurological aggregates. Therefore, manipulating PLCγ2 activity either up or down could have therapeutic benefit; however, we require a better understanding of the signaling pathways propagated by these variants before such clinical utility can be realized. Here, we review the signaling roles of PLCγ2 in hematopoietic cells to help understand the effect of mutations driving immune disorders and cancer and extrapolate from this to roles which may relate to protection against neurodegeneration.

Phosphatidylinositol-specific phospholipase Cγ2 (PLCγ2) belongs to a group of intracellular enzymes that cleave the membrane phospholipid phosphatidylinositol-4,5-bisphosphate (PIP_2_) into diacylglycerol (DAG) and inositol 1,4,5-trisphosphate (IP_3_) (1) (Fig. 1*A*). IP_3_ and DAG then facilitate secondary signaling for membrane-bound immunological and growth factor receptors (1, 2), including activation of PKC and intracellular calcium release (2, 3). PLCγ2 was first cloned in a functional form, including the promoter region, in the hematopoietic Raji cell line (4). PLCγ2 has since been revealed to be involved in a plethora of normal cellular signaling functions in the majority of cells in the hematopoietic system, bone marrow (BM) niche, and bone remodeling as well as various other roles in tissue development and function.

**Figure 1:**
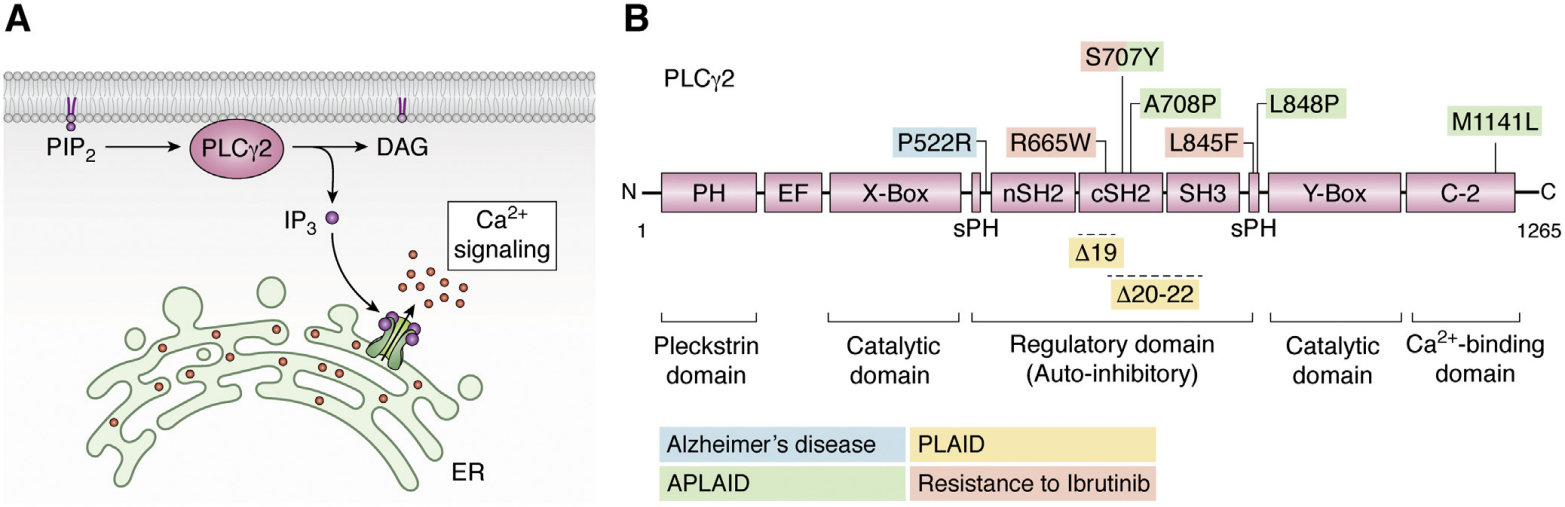
**Activity, structural domains, and known mutations of PLCγ2**. *A*, PLCγ2 belongs to a group of intracellular enzymes that cleave the membrane phospholipid phosphatidylinositol-4,5-bisphosphate (PIP_2_) to diacylglycerol (DAG) and inositol 1,4,5-trisphosphate (IP_3_), resulting in increased calcium signaling. *B*, PLAID-causing genomic deletions (Δ19 and Δ20–22) and APLAID-associated somatic mutations are located within the regulatory domain (S707Y, L848P, A708P) or in the C2 domain (M1141L) of PLCγ2. Acquired mutations as a result of BTK inhibition (S707Y, L845F) and the protective P522R variant in Alzheimer’s disease are found in the regulatory domain of PLCγ2. APLAID, PLCγ2-associated antibody deficiency and immune dysregulation with autoinflammation; C, carboxyl terminus; N, amino terminus; PLAID, PLCγ2-associated antibody deficiency and immune dysregulation; PLCγ2, phosphatidylinositol-specific phospholipase Cγ2.

Structurally, human PLCγ2 is characterized by a multidomain insert between the X box and Y box, which consists of a split pleckstrin homology domain, N-terminal SH2 domain, C-terminal Src homology 2 (cSH2) domain, and SH3 domain (Fig. 1*B*). Interaction between the cSH2 domain and residues surrounding the catalytic active site results in autoinhibition of PLCγ2. Upon Y759 phosphorylation, the cSH2 domain is removed from the catalytic active site and interacts with phosphorylated residue, allowing the substrate PIP_2_ access to the active site (4). Biochemically, PLCγ2 is downstream of many signaling molecules including the kinases Syk Btk, and Lyn, the guanine nucleotide exchange factors Vav, and the GTPase Rac2 that will be discussed in this review. The split pleckstrin homology domain of PLCγ2 (Fig. 1*B*) is essential and sufficient for activation by Rac2, with the crucial amino acids at the PLCγ2–Rac2 interface determined by NMR spectroscopy (5) and protein crystallization (6). Rac2-mediated PLCγ2 activation requires stable translocation to the plasma membrane, and it appears that this proximity to the membrane releases PLCγ2 from autoinhibition (7).

PLCγ2 is also a known risk factor and an important driver in a multitude of diverse disease circumstances including those with an immunological basis such as inflammation, autoimmunity, immunodeficiency, and allergy, as well as in hematological malignancies (Fig. 2). Moreover, research has also suggested PLCγ2 may have a role in Alzheimer’s disease (AD) and some solid cancers. However, the links between PLCγ2’s critical biochemical functions and its diverse biological impacts are poorly understood. Gaining insight into relevant pathways will be important in considering causes of and treatments for a diverse range of diseases. In this review, we describe the current understanding of PLCγ2’s roles in the adaptive and innate immune systems and explore open questions in the field as to how these roles relate to disease pathology. We hope that this is a timely and useful review for researchers interested in learning about how their pathway of interest may be a key avenue involved in unexpected pathological situations.

**Figure 2:**
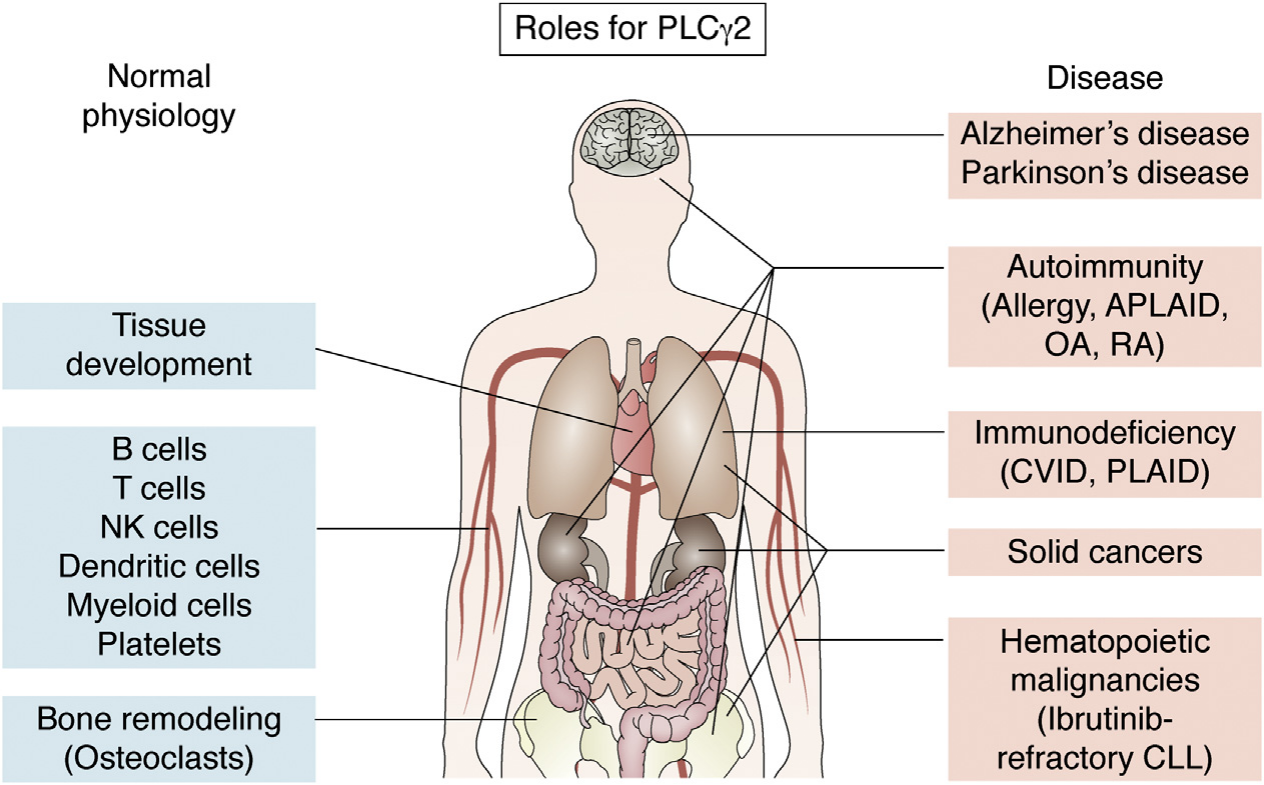
**Organs and tissues influenced by PLCγ2 in health and disease**. An overview of the involvement of PLCγ2 at various anatomical sites in both normal physiology (*left*) and disease settings (*right*) in humans. PLCγ2, phosphatidylinositol-specific phospholipase Cγ2.

## PLCγ2 in the adaptive immune system

PLCγ2 plays a fundamental role in development of mature B cells as a component of the signal transduction cascade that occurs when a B cell receptor (BCR) is stimulated by its cognate antigen. Early studies of PLCγ2 function, typically conducted in the chicken B cell line DT40, revealed that BCR signaling through PLCγ2 required its association with other signal transduction molecules including Syk and Btk in a complex located in a lipid raft, or signalosome. PLCγ2 would then be phosphorylated at four tyrosines (Y753, Y759, Y1197, and Y1217 (8, 9, 10, 11)). Moreover, when maximally stimulated, Y1217 was capable of a 3-fold greater level of phosphorylation than residues Y753 and Y759 in murine splenic B cells potentially demonstrating a potent and unique function of a specific critical tyrosine residue within PLCγ2 (10). In addition to the production of IP_3_, DAG, and calcium flux, PLCγ2 activation results in activation of phospholipase D and cell spreading (12, 13, 14, 15, 16, 17), bringing on other vital cell signaling processes such as Erk (18). Studies using DT40 cells showed that Rac2 could also stimulate PLCγ2 to enhance the BCR-induced calcium flux (19). The BCR coreceptor CD19 is also a vital component of the BCR/PLCγ2 signaling complex requiring phosphorylation of its Y391 residue, which is costimulated with the BCR in response to antigen engagement (20). Upon BCR signaling, PLCγ2 also interacts with B cell adaptor protein with ankyrin repeats (21). PLCγ2 immunoprecipitates with CD19, in a signaling complex with Lyn, Vav, Grb2, and p85 of PI3K, potentially *via* their SH2 domains, suggesting a tight multimeric association of these molecules and with the cell membrane (22).

In addition to this crucial role in BCR signaling, PLCγ2 is also required for the signaling of CD72, a regulatory molecule important in B cell development, although it is thought this process relies on Btk instead of Syk (23). The use of Btk- or Blnk-deficient mice showed their activation was necessary for PLCγ2 signaling (24). BLNK was also noted as being specifically required in human B cells *in vitro* for PLCγ2 phosphorylation and resultant calcium flux after BCR stimulation (25).

The functional requirement of PLCγ2-mediated signaling in B cells was first addressed in mice engineered to lack PLCγ2 enzymatic activity, through the replacement of the second exon (encoding the enzymatic function) with a neomycin cassette (24). Unlike the germline deletion of PLCγ2 that results in embryonic lethality, mice lacking PLCγ2 enzymatic activity were viable, but with profound defects in B cell development and function. These mice exhibited reduced mature B cells, disrupted differentiation of pro-B cells, and a lack of B1 B cells (24). As expected, BCR stimulation failed to induce calcium flux or mitogenic stimulation of proliferation in B cells and serum immunoglobulin (Ig) M, G2a, and G3 and T cell–independent antibody production was reduced in the absence of PLCγ2 function (24). Mice were developed where the exon of PLCγ2, bearing the catalytic region containing the PIP_2_-binding site, was floxed and inducibly deleted in adult mice (26). Indeed, PLCγ2-deficient and inducible PLCγ2^−/−^ mice have been widely shown to lack the ability to respond to antigens (24, 26, 27, 28).

The importance of BLNK to PLCγ2 function during B cell development was revealed through the use of BLNK^−/−^PLCγ2^−/−^ mice where floxed enzymatic PIP_2_ domains of PLCγ2 were inducibly deleted (26, 29). BLNK/PLCγ2 double-deficient mice showed a more pronounced defect in early B cell progenitors from around the Pro-B cell stage than in the single KO of BLNK or PLCγ2 (29). This study also showed that PLCγ2 dosage was important as PLCγ2 biochemical function and B cell development were reduced in the PLCγ2 heterozygous mice on a BLNK-deficient background, compared with BLNK^−/−^PLCγ2^+/+^ mice (29). It is worth noting that PLCγ2 concentration is not constant through B cell ontogeny, as immature B cells express 3-fold more PLCγ2, as well as BLNK and Btk, leading to enhanced BCR-mediated phosphorylation of PLCγ2 (30). Significantly higher levels of PLCγ2 were also observed in human IgM memory and switched B cells than naïve B cells (31).

The first PLCγ2^−/−^ mouse studies showed a block in B cell maturation from transitional 2 to follicular B cells, with these cells also exhibiting increased levels of BCR-induced apoptosis (28, 32). The importance of PLCγ2 was additionally shown in terms of light-chain loci activation, required for recombination and receptor editing of autoreactive B cells (28). B cells in these PLCγ2^−/−^ mice showed a downregulation of the potent prosurvival protein Bcl-2 and impaired BCR-induced expression of A1, another prosurvival Bcl2 family member (32). Bcl-2 overexpression however did stop BCR-induced apoptosis in all splenic B cells and partially restored numbers of follicular B cells (32). The incomplete nature of the rescue of PLCγ2 deficiency by Bcl2 overexpression was due to Bcl2 not being able to compensate for an additional function of PLCγ2 in mediating B cell–activating factor receptor signaling, which is also necessary for B cell survival and maturation (33).

Broader regulation of PLCγ2 function may involve several transcription factors, such as Ikaros, which was shown to be essential in generating B cells, but not T cells, in mice (34, 35, 36). Deletion of Ikaros in the DT40 cell line also showed its absence impaired BCR signaling *via* PLCγ2 and associated calcium flux, suggesting a role of Ikaros in functional control of PLCγ2 (37). In addition, transcription factors, NFAT and AP-1, are also induced after BCR stimulation in B cells mediated by PLCγ2 (38). Enzymatic deficiency of PLCγ2 also downregulated BCR-induced upregulation of IRF4 and IRF8, both vital transcription factors for rearrangement process of λ and κ light chains (28). Collectively, these studies reveal that PLCγ2 is an important component in several immunological pathways resulting in the induction of key transcription factors necessary for subsequent immunological functions.

Several biochemical pathways and processes were shown to negatively regulate PLCγ2 activity and the resulting calcium flux after BCR engagement. These include RasGRP3 (39, 40), Bam32 (41), hematopoietic adaptor protein Dok-3 (downstream of kinase-3) with Grb2 (42), Themis2, an adaptor protein with no known enzymatic activity, *via* constitutive binding to Lyn, Grb2, and PLCγ2 to mediate its function (43), BLNK *via* impairing its association with the C2-phosphotyrosine domain of PLCγ2 (44), and SFRP2 (secreted frizzled-related protein 2), a potential Wnt inhibitor (45). BCR signaling, *via* Syk, BLNK, PLCγ2, and NF-κB, is also inhibited *via* BTLA (B and T lymphocyte attenuator) associating with the BCR and *via* binding its known ligand, herpes virus entry mediator and activating SHP-1 (46). Activation of PLCγ2 and subsequent calcium flux is also negatively regulated by the E3-ubiquitin protein ligase isoforms c-Casitas B-lineage lymphoma (Cbl) and Cbl-b (47, 48, 49, 50). B cell double c-Cbl and Cbl-b KO studies in mice resulted in a systemic lupus erythematosus (SLE)-like autoimmune disease (50). Engagement of the BCR in these mice resulted in enhanced PLCγ2 signaling, including Syk and calcium flux, but reduced BLNK and no detectable PLCγ2 ubiquitination, a known function of Cbl (50, 51). Examining the pre-B cells of mice lacking both c-Cbl and BLNK, compared with those with one allele of c-Cbl, but still in the absence of BLNK, revealed elevated levels of Btk and PLCγ2 (51). The plethora of negative regulators suggested to help control BCR/PLCγ2/calcium flux signaling within B cells underscores the importance of tight regulation of this cellular process in mediating immune responses, without which would very likely further contribute to development of inflammatory disease.

Aside from BCR engagement, there are alternative signaling pathways initiated through extracellular immunological receptors that also utilize PLCγ2 to function, which include CD38 (16), CD40 (52), IL-4R (53, 54, 55) and the LPS/TLR4/MyD88 pathway, the latter of which induces ERK and NK-κB phosphorylation in MEKs in a PI3K-independent fashion (56). CD86 engagement was also observed to signal *via* PLCγ2 inducing Oct-2 expression (57, 58). TGF-βR stimulation has also been shown to inhibit PLCγ2 function in B cells (59). These alternative extracellular receptor signaling pathways reveal the utility of PLCγ2 in delivering important cellular messages for B cells to mediate a range of immunological functions.

The role of PLCγ2 in the immunological functions of T cells is comparatively more limited than that of B cells, but it still serves important functions in certain cellular contexts. Inducible KO mice showed T cell development and T cell receptor (TCR)-mediated proliferation were normal in the absence of PLCγ2 (24, 26), with development instead depending on the closely related family member PLCγ1 (60). However, PLCγ2 is required in primary T cells, in association with a linker for activated T cells and SH2 domain–containing leukocyte protein of 76 kDa (SLP-76), when signaling upon TCR stimulation, with PLCγ1/2 double-mutant cells showing impaired TCR signal transduction with regard to calcium flux and subsequent Erk activation compared with the PLCγ1 KO alone (61). Double deficiency for PLCγ1/2 also has a more severe T cell developmental phenotype, particularly in the transition of CD4/CD8 double-positive thymocytes to the CD4 or CD8 lineage (61). The calpain-calpastatin system is a fundamental part of membrane fusion events mediated by the proteolysis of amyloid precursor proteins which is an important process for effective responses to antigenic challenges. In resting human T cells, the calpain-calpastatin system, specifically calpain inhibition, also played a role for PLCγ2 phosphorylation and the subsequent calcium flux, along with the phosphorylation of p56Lck and NF-κB (62).

In summary, PLCγ2 plays a fundamental role in B cell development affecting the survival of mature B cells and antibody production, as well as being an absolutely necessary and immediately proximal component of the BCR signaling cascade. While PLCγ1 plays a greater role in T cells, PLCγ2 is still important in terms of TCR signaling and the calpain-calpastatin system and when combined with PLCγ1 deletion. Considering these vital roles for PLCγ2 in the adaptive immune system, it is of little surprise its dysregulation or malfunction is also then of crucial importance in a number of disease settings of an immunological and hematological nature.

## PLCγ2 and immune deficiency

Given its prominent role in the adaptive immune system, PLCγ2 has been implicated in manifestations of immunodeficiency, including both the immunodeficiency syndrome, PLCγ2-associated antibody deficiency and immune dysregulation (PLAID) and common variable immunodeficiency (CVID). PLAID is specifically driven by a gain-of-function amino acid mutation in the PLCγ2 gene (63, 64), whereas CVID is much more genetically diverse with 2% of published cases of CVID estimated to be due to mutations in the *PLCG2* gene (65). Patients with PLAID typically exhibit symptoms of cold urticaria, a form of cold temperature-induced hives, beginning in infancy, but otherwise shares many of the common clinical manifestations of CVID such as granulomatous disease, allergy, autoimmune disease, and an abnormally low concentration of serum IgG with recurrent infections (63, 66). The cellular infiltrate of the skin granulomas observed in patients with PLAID is neutrophilic and monocytic in nature, as a result of leukocyte hyperactivation, making it histopathologically very similar to that seen in CVID (67). Chemotactic ability of neutrophils from patients with PLAID was also noted to be defective *in vitro* (67). Patients with PLAID also typically have suboptimal numbers of NK cells, low serum IgM and IgA, weak vaccination responses, and low frequency of circulating switched memory B cells (68).

The biochemical mechanism behind the PLAID phenotype may be at least partially explained by examining the role of the cSH2 domain of PLCγ2, which is affected in patients with PLAID (69). *In vitro* studies revealed that after BCR signaling, the cSH2 domain of PLCγ2, coupled with poor phosphorylation of Syk, Btk, and BLNK, stabilized the early signaling complex (69). This in turn resulted in reduced clustering with the BCR and impaired downstream signaling (69). The authors of this study concluded that the impaired cellular movement of the antigen-engaged BCR may be responsible, at least in part, for the PLAID syndrome through reduction of specific antigen engagement by T and B cells that are required to activate and differentiate B cells allowing antibody secretion and formation of B cell memory (69). A common symptom of patients with PLAID is cold-induced urticaria, which may also be linked to mutations in the SH2 region of PLCγ2 as the lack of that region resulted in over a 100-fold rapid increase in activation status after only a few degrees Celsius of cooling but was distinct from a lack of autoinhibition (70).

Overall, these studies of CVID and PLAID lay bare the crucial role of PLCγ2 in the etiology of these diseases because of PLCγ2’s underlying role in the vital immunological signaling of lymphoid cells. Further research into these immunological diseases involving PLCγ2 will help reveal much needed therapeutic inventions.

## PLCγ2 in the innate immune system

PLCγ2 is also very important in the context of myeloid cells including monocytes, macrophages, NK cells, dendritic cells (DCs), and mast cells often because of its utility in facilitating downstream signaling after FcR engagement. Moreover, PLCγ2 is reported to be involved in essential signaling processes associated with integrin activation and signaling downstream of critical myeloid receptors including cytokine receptors and TLRs. This signaling drives subsequent antibacterial functions including degranulation and production of reactive oxygen species (ROS) and inflammatory responses.

In monocytes, macrophages, and mast cells, PLCγ2 is activated through crosslinking of the FcεR and FcγR (71). While PLCγ2 KO mice have normal numbers of myeloid cells, and mast cells exhibit normal MAPK activation and cytokine mRNA levels, they show impaired FcεR-stimulated calcium flux, IP_3_ production, degranulation, and inflammatory cytokine release (71). FcγR meanwhile is normal in macrophages and monocytes, but PLCγ2-deficient mice are resistant to IgE-mediated cutaneous inflammatory skin reaction (71). Macrophages signaling through FcγRI and FcγRIII express both SLP-76 and BLNK, which can phosphorylate PLCγ2 (72). PLCγ2 is also essential in neutrophils for β-2 integrin and FcγR-mediated effects including production of ROS, degranulation, and cell spreading (73). PLCγ2 was also shown to be necessary for ROS production in the neutrophil response to inflammatory microcrystals (74). Research exploring the CD16b (FcγRIII) inhibitor LFM-A13 in human neutrophils also revealed that CD16b signals through Btk/PLCγ2 and associated calcium flux (75), although PLCγ2-deficient mouse neutrophils respond normally to agonists including chemokines, bacterial formyl peptides, TLR ligands, and proinflammatory cytokines (73).

The Fms (M-CSF) receptor, essential for macrophage development, also associates with PLCγ2, leading to its activation, resulting in PI3K activation, thus showing a potential role in myeloid cell differentiation (76, 77). Blocking of either Src family kinases (78) or PLCγ2 directly showed it was required downstream of M-CSF signaling to facilitate human monocyte differentiation (79).

In addition to FcR signaling requiring PLCγ2 for efficient ROS production in murine neutrophils, PLCγ2 is also essential for postintegrin signaling induction of ROS, as well as being critical for the neutrophil-dependent host defense against bacterial infections *in vivo* (80). Murine macrophages lacking PLCγ2 are also defective in the pattern recognition receptor for fungal cell walls, dectin-2 signaling in response to fungal infections as highlighted by reduced inflammatory cytokine responses, NF-κB and MAPK signaling, ROS, and clearance of fungal infections *in vivo* (81). PLCγ2 can also be exploited by some intracellular bacteria, such as *Ehrlichia chaffeensis* in the monocytic THP-1 cell line, where the recruitment and activation of PLCγ2 is required to maintain stable calcium levels to facilitate intracellular infections (82). PLCγ2 was also found to be phosphorylated (at Y1217) by *Mycobacterium tuberculosis* (the causative agent of tuberculosis (TB)) in the macrophage cell lines (83). Knockdown of PLCγ2 enhanced TB uptake and killing *via* NO and blocked the TB-mediated inhibition of proinflammatory cytokine production including TNF-α and RANTES (83). These studies underscore the importance of PLCγ2 activation for the survival of intracellular bacteria.

The use of BM-derived macrophages and DCs from PLCγ2 KO mice showed that PLCγ2 was required after peptidoglycan/TLR2 and LPS/TLR4 stimulation to induce normal levels of TNF-α and IL-6 (84). siRNA and inhibitor studies in a macrophage cell line further highlighted the requirement of PLCγ2 in LPS-induced TLR4 signaling in terms of IP_3_ production calcium flux and endocytosis, as well as downregulation of IRF3, but with the increase of NF-κB signaling (85). The activity of both PLCγ2 and PLCγ1 is triggered in primary macrophages in response to titanium wear particles (generated as a consequence of the degradation of titanium arthritic joint replacements) *in vitro*, suggesting an advantage to inhibiting PLCγ2 in the context of joint replacement (86). PLCγ2 also has a role in neutrophil recruitment *via* Btk in regulating E-selectin–mediated integrin activation, which facilitates leukocyte rolling and infiltration into inflamed tissues, and subsequent PI3Kγ pathways (87). Indeed, PLCγ2 phosphorylation, along with Syk, Atk, and p38 MAPK, is required for successful E-selectin–mediated slowing of leukocyte rolling and subsequent transmigration (88). DCs also use PLCγ2 for signaling of major histocompatibility complex (89), integrin receptors, TLRs, and the dectin-1 pathways in a manner similar to macrophages regulating DC migration (90, 91) and antifungal immunity (92).

NK cells also use PLCγ2 during their development and to elicit various cellular functions including CD69 signaling, secretion of cytotoxic granules, response to infection, and tumor immunosurveillance. CD69-mediated cytotoxicity of human NK cells has been shown to signal through Syk/Src family tyrosine kinase and PLCγ2/Vav1 (93). Indeed, PLCγ2 is essential for murine NK cell cytotoxic function as it is required for cytotoxic granule secretion and calcium flux (94, 95). This lack of cytotoxicity in the absence of PLCγ2 in NK cells resulted in the failure of NK cell–mediated rejection of major histocompatibility complex I–negative lymphoma cells and NK cell–mediated control CMV infection *in vivo* (94, 95). The PLCγ2-deficient mouse also revealed a defect in lineage-committed NK precursor cell expression of Ly49 receptors, which impaired the terminal maturation of NK cells (96).

A number of studies also noted that PLCγ2 is required for osteoclastogenesis and within the BM niche. Using colony assays and a PLCγ2 inhibitor in both mouse and human hematopoietic stem and progenitor cells, it was found that PLCγ2 is responsible for IL-3 and GM-CSF-induced signaling through the resultant calcium flux, IP_3_ production, and subsequent MEK/ERK activation (97). In megakaryocytes, PLCγ2 is also a crucial downstream signaling molecule, along with Syk and SLP-76, for the surface protein CLEC-2 (98). CLEC-2 in turn regulates the production of thrombopoietin that is essentially required for the BM niche and the proper maintenance of hematopoietic stem cells (98).

Receptor activator of nuclear factor kappa-B ligand (RANKL) is a fundamental cytokine governing bone formation and destruction *via* the RANK receptor expressed on osteocytes. RANKL-induced osteoclastogenesis requires PLCγ2, which controls downstream functions including NFATc1, AP1, and NF-κB (99). In contrast, another study reported that PLCγ2-deficient mice showed impaired RANKL-induced activation of MAPK, p38, and JNK but not ERK and NF-κB, AP-1, and NFATc1 (100). They also noted a failure of PLCγ2-deficient BM macrophage precursors to differentiate into osteoclasts after RANKL stimulation, which could still be rescued with PLCγ2 and not PLCγ1 (100). Moreover, PLCγ2-deficient mouse studies showed that blocking PLCγ2 impedes the early development and function of osteoclasts, thereby preventing bone loss in arthritis (99, 100). This effect was independent of PLCγ1 (99). Another group also observed that PLCγ2 was required in the regulation of integrin-mediated osteoclast cell adhesion migration and resorption of the bone (101). Ephrin A1 reverse signaling, a process responsible for bone resorption, may also be mediated by PLCγ2 (102). These numerous reports give great scope to the incredibly important innate immune functions that require PLCγ2 in their execution and exemplify why the corruption of these processes is of great relevance for inflammatory disease, as now discussed below.

## PLCγ2 and allergy

Mast cells are one of the primary mediators in allergic responses. They are activated *via* engagement of their antigen-specific IgE receptors that in turn release their proinflammatory payload. PLCγ2 is implicated in allergic responses, specifically with regard to the function of mast cells and their crosslinking of FcεRI (IgE-binding receptor) and FcγR and subsequent degranulation (71). Studies of PLCγ2-deficient mice revealed they had normal numbers of mast cells but impaired FcεR-stimulated calcium flux, IP_3_ generation, degranulation, and cytokine responses despite normal MAPK activation and cytokine mRNA levels (71). The PLCγ2-deficient mice were also resistant to IgE-mediated cutaneous inflammatory skin reactions (71). By way of a potential therapy, dexamethasone was found to inhibit mast cell inflammatory mediators by blocking FcεRI-mediated PI3K activation *via* downstream signaling molecules Grb2 and PLCγ2, and associated calcium flux, by blocking degranulation and the production of inflammatory cytokines (103). Targeting the transcription factor Zeb2 using siRNA in mast cells revealed it may regulate Btk/PLCγ2, as evident by their decreased expression, which impacted signal transduction *via* the FcεRI∖Grb2∖PLCγ2 pathway, which was in turn responsible for the production of inflammatory mediators (104). And another study in mast cells, in which the signaling adaptor protein T cell ubiquitin ligand-2 was knocked down, showed it, like SHIP-1, functions as a negative regulator of FcεRI signaling *via* Syk/PLCγ2 phosphorylation and the resultant degranulation (105). These studies collectively show that targeting PLCγ2 may have benefits for the treatment of diseases associated with an overreactive immune response.

## PLCγ2 and autoinflammatory disease

Autoinflammatory diseases are characterized by recurrent episodes of systemic inflammation, skin lesions, and arthritic joints. Understanding the mechanisms behind the role of PLCγ2 in autoinflammatory diseases was enabled by the identification of the *Ali5* mice that bear a gain-of-function point mutation resulting in a single amino acid change (D993G) in PLCγ2 resulting in spontaneous inflammation (106). These mice are characterized by an expansion of innate immune cells, T/B cell–independent inflammation, hyperactive calcium flux in their B cells, and formation of autoantibody complexes (106). Further work utilizing N-ethyl-N-nitrosourea alkylating mutagenesis resulted in the creation of the *Ali14* mutant mice, in which gene mapping revealed a Y to C missense mutation in PLCγ2 at amino acid position 495 within the SH2 domain (107). These mice exhibited a dominantly inherited disease characterized by spontaneous hind paw swelling/inflammation (107). Hyperactive calcium flux was observed in their B cells, with an abnormally high T:B cell ratio, especially in the peripheral blood, as well as increased serum Ig, cholesterol, and triglycerides (107). Male mice presented with increased IgG1 and IgG2b, whereas serum IgM was elevated in both sexes (107). *Ali14* mutant mice also exhibited changes to their body composition with reduced fat mass and a lesser bone mineral content, the latter suggesting an impact on osteoblastic functions (107).

**Figure 3:**
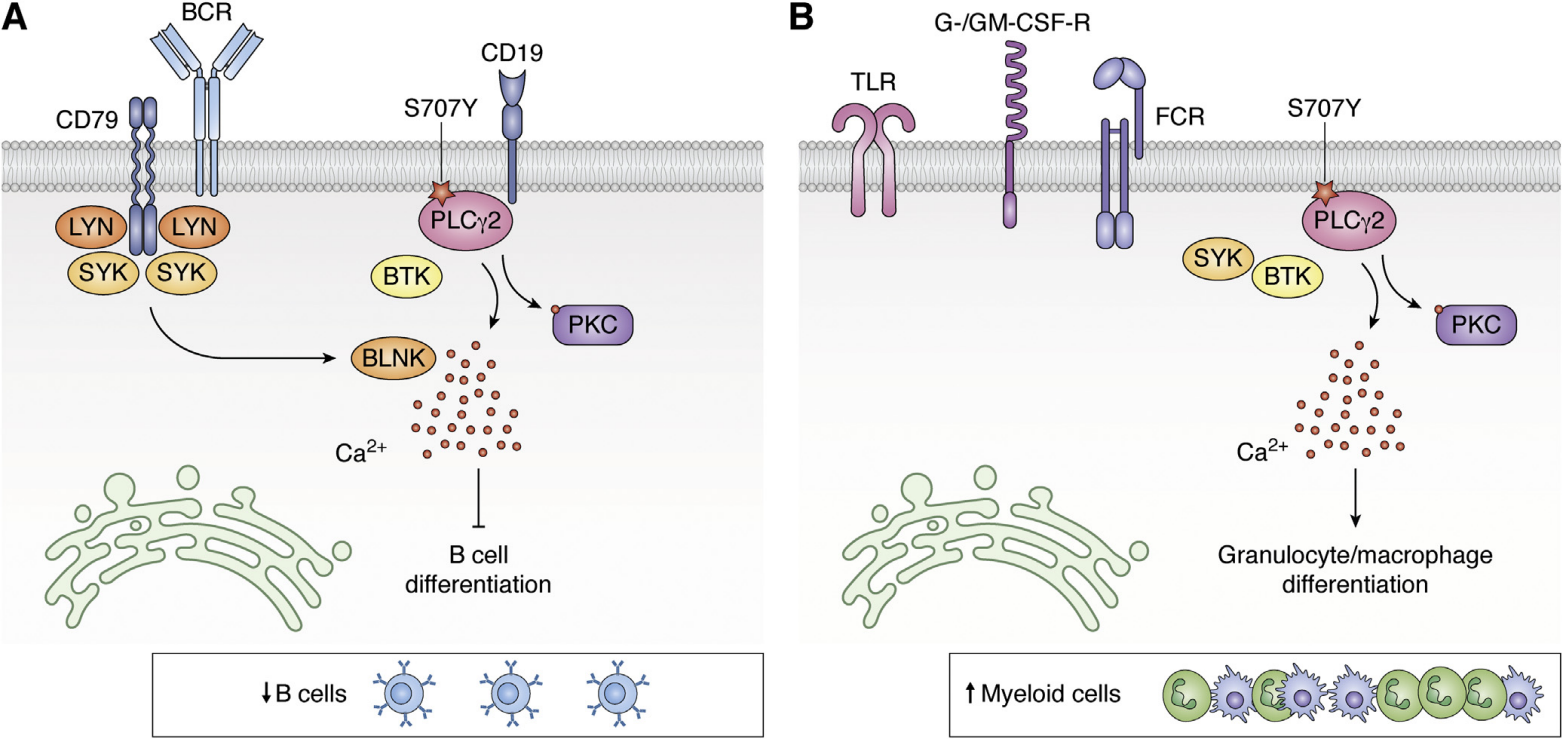
**Opposing cellular effects of PLCγ2 mutations in APLAID**. In APLAID, PLCγ2 mutations result in (*A*) stronger BCR signaling including Syk, Btk, and BLNK and elevated calcium responses and therefore negatively impact B cell development and (*B*) increased production of granulocytes and macrophages, which possibly depends on an increased GM-CSF receptor signaling. APLAID, PLCγ2-associated antibody deficiency and immune dysregulation with autoinflammation; PLCγ2, phosphatidylinositol-specific phospholipase Cγ2.

In humans, seven cases of autoinflammation caused by PLCγ2 gain-of-function point mutations, summarized under the acronym APLAID, have been reported (69). These patients exhibit both phenotypic differences and similarities with PLAID, including involvement of the humoral immune (Fig. 3*A*) and innate system (Fig. 3*B*) as well as various forms of inflammation (64). Common clinical features in APLAID include early-onset, recurrent blistering skin lesions, pulmonary disease, arthralgia, inflammatory eye and bowel disease, and mild immunodeficiency (64). Laboratory analysis of patients with APLAID showed decreased IgM, IgG, and IgA levels, typically lacking class-switched memory B cells, with normal NK and T cell counts. There are no standard treatment guidelines for APLAID’s management. Patients were refractory to treatment with TNF-α inhibitors and responded partially to the IL-1 receptor antagonist Anakinra (108).

Mechanistically, the pathogenesis in patients with APLAID is not yet well understood. The inflammation found in APLAID has partially been attributed to the activation of the NLRP3 inflammasome as patient’s blood mononuclear cells secreted increased levels of IL-1β (109). In contrast, whole-blood assays revealed a reduced production of IL-10 and IL-1β after LPS stimulation (110).

Most mutations found in APLAID lie within the autoinhibitory domain (Fig. 1), causing failure of autoinhibition and resulting in constitutive phospholipase activity resulting ultimately in an increased production of both intracellular IP_3_ and calcium. The C2 domain of PLCγ2, in which the APLAID mutation M1141L resides, has previously been shown to be critical for calcium-dependent translocation of PLCγ2 to the plasma membrane for the sustained influx of extracellular calcium. It has been proposed that as this mutation is proximal to the calcium-binding site, it may result in higher affinity binding, leading to increased IP_3_ and DAG production. As a consequence, binding of the SH2 domain to BLNK is also stabilized by the C2 domain in a calcium-regulated manner (44).

## PLCγ2 and autoimmune disease

PLCγ2 also appears to play a role in the chronic inflammatory disorder, rheumatoid arthritis (RA), as evident by an upregulation of the PLCγ2 gene signature of patient peripheral blood mononuclear cells (111). Indeed, PLCγ2 was revealed to be involved in RA-related gene pathways such as inflammasome activation and both platelet aggregation and activation, which makes it a promising drug target to stem inflammation in RA (111). In a K/BxN serum-transfer murine model of RA, Vav/PLCγ2-deficient mice were protected from inflammation and bone erosion (73, 112). This involvement of PLCγ2 was downstream of integrin stimulation and neutrophil activation, including their spreading and degranulation (112). B cell adaptor protein with ankyrin repeats is a known risk factor in autoimmunity including SLE and systemic sclerosis (113, 114). Indeed, patients with SLE saw clinical benefit through treatment with an anti-CD22 mAb therapy, Epratuzumab (115). The potential mode of action in human B cells by Epratuzumab was observed to be elicited by a downregulation of Syk/PLCγ2 signaling, including calcium flux, after BCR stimulation (116). Thymocyte-expressed, positive selection–associated 1 is critical in thymic T cell development and activation of mast cells *via* FcεRI, as well as being of importance to B cell functions including proliferation, activation, germinal center formation, and serum antibody production (117). Pathogenic B cells which form in the thymocyte-expressed, positive selection–associated 1 KO mice in a collagen-induced arthritis model were impaired in PLCγ2 function, and associated calcium flux, thereby reducing the severity of their disease in comparison with control mice (117).

Lyn tyrosine kinase autoactivation (gain-of-function) mice, after BCR stimulation, showed constitutive phosphorylation of Syk/PLCγ2, with a resultant increased calcium flux, observed in their resting B cells (118). These circumstances of PLCγ2 hyperactivation led to an increased level of autoantibodies in these mice and ultimately a lethal autoimmune glomerulonephritis (118). Common genetic variants in *PLCG2* also link the enzyme to type 1 diabetes (119) and venous thromboembolism (120), which could have a shared autoimmune etiology.

Overall, PLCγ2 clearly exhibits an important role in the manifestation of autoinflammatory disease with hypermorphic PLCγ2 signaling resulting in spontaneous autoinflammatory disease. These PLCγ2 signaling-induced autoimmune diseases produce a range of inflammatory phenotypes in both patients and disease models, typically driven by autoantibodies (with the exception of APLAID) and broad autoimmune cell expansion. These disease situations highlight the potential to develop PLCγ2-modulating therapies that treat immune disease but must be tailored so as to avoid effects on other disease relevant processes such as cancer and neurodegeneration. Next, we explore recent insights and advances in these fields.

## PLCγ2 and cancer

The crucial nature of PLCγ2 in both the innate and adaptive immune system is further underscored by its vital roles in hematological malignancy. This is perhaps most evident in chronic lymphocytic leukemia (CLL), but PLCγ2 is also observed to play a role in diffuse large B cell lymphoma (DLBCL), the most common form of non-Hodgkin’s lymphoma, Hodgkin’s lymphoma, myelodysplastic syndrome, endemic Burkitt lymphoma (EBL), MALT-associated gastric lymphoma and multiple myeloma. Finally, PLCγ2 has also been shown to be a relevant part of Epstein–Barr virus (EBV) transformation of B cells.

A powerful tool for validating the roles of oncogenes and tumor suppressors is the Eμ-Myc mice lymphoma model where all mice will succumb to lymphoma. Using this Eμ-Myc lymphomagenesis mouse model in the absence of PLCγ2 demonstrated that PLCγ2 was indeed vital to early B cell development, resulting in an accumulation of large pre-B cells (121). PLCγ2 deficiency affects the functionality of pre-BCR, resulting in increased IL-7 signaling and upregulation of the RAG recombinase in selected large pre-B cells, thus leaving them vulnerable to transformation and accelerating c-myc-mediated lymphomagenesis (121).

While the absence of PLCγ2 can further enable lymphogenesis in certain murine lymphomas, in humans, PLCγ2 signaling can drive leukemogenesis in some cases. Ibrutinib, which inhibits Btk and thus blocks PLCγ2 signaling, has become an important and effective treatment for CLL. However, a subset of patients become refractory to this treatment over time and relapse, with PLCγ2 as a primary driver of the recurrent disease (122, 123). Mutations in PLCγ2 and/or Btk have been reported in 11 to 90% cases of Ibrutinib-refractory CLL, further underscoring the importance of better understanding the process by which the partnership of Btk and PLCγ2 drives CLL disease progression (122, 123, 124, 125, 126). Interestingly, two separate studies observed that there were no PLCγ2 or BTK mutations detected in patients with CLL before Ibrutinib treatment (127, 128). However, after Ibrutinib treatment, it was observed that CLL progression favored the selection of rare subclones bearing PLCγ2 mutations (129, 130). Collectively, these studies speak to the significant merit of monitoring patients with CLL for development of PLCγ2 mutations after their Ibrutinib treatment. This knowledge may be beneficial to clinicians by serving as an early clinical indicator of likely relapse and potentially help inform a more prudent treatment course for the patient.

Hypermorphic gain-of-function genetic changes in the putative calcium-regulated domain (C2) of *PLCG2* were noted to be correlated with CLL resistance to Ibrutinib (131). It was also demonstrated *in vitro* SYK and LYN were essential in the mutated hypermorphic PLCγ2 (R665W) signaling, a common relapse patient PLCγ2 mutation, suggesting SYK and LYN as alternative targets in Ibrutinib resistance (132). SYK/PLCγ2 activity is also suggested as a potential biomarker for responsiveness in the treatment of CLL and DLBCL, as their signaling activity correlated with cell death induced by treatment with the Src tyrosine kinase inhibitor, Dasatinib (133, 134). Furthermore, PLCγ2 mutations R665W and L845F in Ibrutinib-resistant CLL were shown to be hypersensitive to the Rho GTPase, Rac2 protein *in vitro*, which may suggest Rac2 as a potential target of refractory disease (135).

PLCγ2 has also been implicated in 63% of DLBCL cases (54 of 86 patients in the study) (136). Treatment of these patient cells with an inhibitor of PLC (U73122) *in vitro* suppressed their proliferation and induced apoptosis and cell cycle arrest (136). However, unusually, high PLCγ2 expression was actually associated with better overall survival, which the authors speculated that potentially the stronger PLCγ2 signaling enhancing cellular proliferation and thus making the cells more susceptible to cytotoxic drugs (136). Downregulation of PLCγ2 gene pathways, and other BCR-related genes, along with EBV infection, is also associated with Hodgkin’s lymphoma (137). The use of the *Ali5* heterozygous point mutation, gain-of-function PLCγ2 mice (106) in a model of *Helicobacter felis* infection showed PLCγ2 protects mice from developing gastric MALT lymphoma (138). These mice also had decreased levels of inflammatory cytokines and antibody responses to *H. felis*, suggesting a weakened immune response after infection that the authors ventured was due to the increased presence of regulatory T cells (138). Mutations in the PLCγ2 (Q548R) gene, along with PIK3CD (D133E) and AKT3 (D280G), were implicated in myelodysplastic syndrome progression after the chemotherapeutic combination treatment of Azacitidine/Lenalidomide in the cohort of patients who were either first to become refractory to therapy or failed entirely to respond to therapy (139). These mutations also were noted to correlate with shorter overall survival and duration of response to treatment (139).

In addition to the transformative influence of PLCγ2, in certain the hematological malignancies, PLCγ2 also serves a role in EBV transformation of activated B cells. It was noted that PLCγ2 and its BCR-associated signaling are blocked *via* the virally encoded integral latent membrane protein 2A in combination with BLNK (140). While PLCγ2 signaling is apparently blocked during EBV infection, it was also conversely shown to be upregulated in EBV-transformed lymphoblasts (141). This potentially indicates that after initial EBV infection, PLCγ2 function may be restored in the transformed lymphoblasts. However, it remains to be confirmed beyond this initial observation as to whether this is indeed the process that occurs during EBV transformation and why. In addition, an RNA-seq study of patient tumors of EBL, a common childhood cancer from equatorial Africa where malaria is endemic, showed that the vast majority of cases which harbor EBV contain PLCγ2 mutations, along with mutations in other genes including *MYC* (142). Interestingly, while *MYC* translocation and hyperexpression is a hallmark of EBL, it in itself is not sufficient to induce lymphogenesis (143), which may at least in part explain why mutations in PLCγ2 are relevant to the disease initiation (142).

PLCγ2 is also potentially involved in a small number of solid cancers such as Wilms' tumor (kidney), osteosarcoma, esophageal squamous cell carcinoma, esophageal adenocarcinoma, cervical adenocarcinoma, Birt-Hogg-Dube tumors, non–small-cell lung cancer, and breast cancer. PLCγ2 was implicated in the Wnt signaling pathway necessary for kidney development and development of Wilms' tumor (144). In addition, *PLCG2* was implicated as a potential tumor suppressor gene in Wilms' tumor with a focal deletion of chromosome 16q22.1q24.3, which resulted in a copy number alteration and downregulation of *PLCG2* (145). PLCγ2 was also found to potentially contribute to other tumorogenic situations such as the formation of esophageal adenocarcinoma (146). This is thought to be through PLCγ2’s necessary role in contributing to excessive ROS production and cell proliferation, induced by taurodeoxycholic acid, causing the activation of PI-PLCγ2, ERK-2, and MAPK and production of NADPH oxidase NOX5-S. The resulting mutagenic stress may also be a factor in the development of esophageal adenocarcinoma (146). PLCγ2 was also implicated in cervical adenocarcinoma, where *in vitro* siRNA knockdown of PLCγ2, and calmodulin 1, in human cervical adenocarcinoma cells led to an increased sensitivity to doxorubicin and paclitaxel, but not Cisplatin (147). However, PLCγ2 was noted to be involved in Cisplatin resistance in another study of seven cancer cell lines, suggesting that the influence of PLCγ2 on tumorogenesis may be dependent on the cellular context (148). Population studies and microarray analyses also revealed PLCγ2 associated with the EGFR pathway was a risk factor in esophageal squamous cell carcinoma and gastric cancer (149), in non–small-cell lung cancer associated with HMGB1 expression (150), and as upregulated and potentially involved in triple-negative breast cancer–associated BRAC1 in a Chinese patient cohort (151). Indeed, PLCγ2 SNPs were identified as breast cancer risk factors in patients on menopausal hormone replacement therapy (152, 153).

Using patient material and mouse/human xenograft models, PLCγ2 was revealed to be an overexpressed upstream mediator, among other factors resulting in ERK signaling, that induced increased FGF-mediated bevacizumab resistance in head and neck squamous cell carcinoma (154). siRNA targeting Ezrin, a cytoplasmic peripheral protein involved in metastatic spreading of osteosarcoma and dependent on PIP_2_, also resulted in PLCγ2 upregulation in human osteosarcoma cell lines (155, 156), while another study of osteosarcoma cell lines found PLCγ2 to be a differentially regulated gene (157). PLCγ2 as was also noted as a potential biomarker of radiation exposure through its upregulation after ionizing radiation (158). The detection PLCγ2 in such wide array of solid cancers speak to its great potential as a prognostic indicator and drug target.

Overall, these studies demonstrate that PLCγ2 is a driver or risk factor in a number of human cancers, both solid and hematological. However, in mice, the absence of PLCγ2 can help drive lymphomagenesis. Thus, more research is needed to understand the distinct roles of PLCγ2. Nevertheless, PLCγ2 clearly plays a crucial role in CLL that has become refractory to Ibrutinib treatment. In this context, PLCγ2 serves as a vital biomarker for CLL progression during Ibrutinib treatment in the clinical setting. It is also fascinating that the same mutation can drive both resistance to Ibrutinib and the inherited immune disease APLAID (Fig. 1). Therefore, efforts to therapeutically target the effect of these mutations could be broadly applicable across a range of pathologies.

## PLCγ2 and neurological diseases

**Figure 4:**
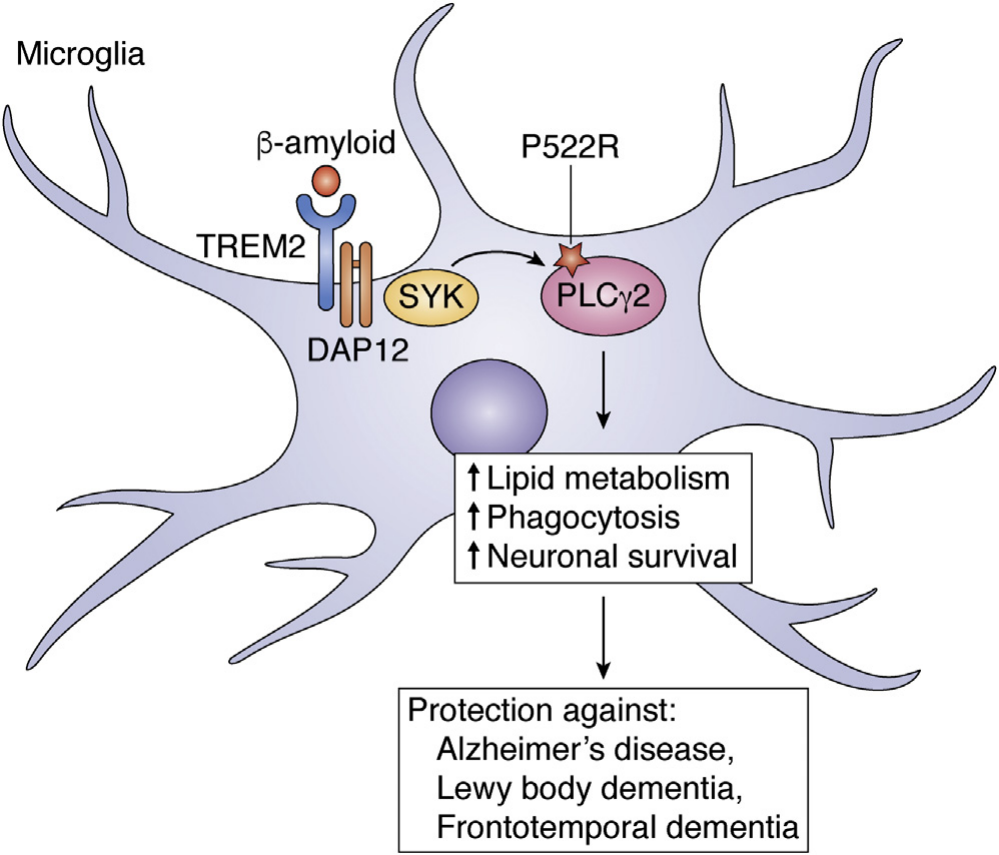
**The PLCγ2 variant P522R is protective against neurodegeneration**. In the brain, the γ2 isoform is predominantly expressed by microglia (*right*). When triggered by TREM2, the PLCγ2 variant P522R supports lipid metabolism, phagocytosis, and survival (*left*), thus protecting against Alzheimer’s disease (AD), Lewy body dementia, and frontotemporal dementia (FTD). PLAID, PLCγ2-associated antibody deficiency and immune dysregulation.

Late-onset AD is the most common form of dementia, characterized by accumulation of amyloid β (Aβ) and neurofibrillary tangles. A rare variant in the *PLCG2* gene (Pro522Arg) has been associated with decreased risk of AD by genome-wide association study (159). The initial genetic results were replicated by various independent cohorts (160, 161, 162). Recently, the reduced risk of the P522R mutation for AD has been extended to other dementia subtypes including Lewy bodies and frontotemporal dementia (163) (Fig. 4). This variant was also associated with an increased chance of reaching at least 90 years of age and then becoming a cognitively healthy centenarian, that is, people in whom the absence of dementia and extreme longevity is combined (163). In contrast, the PLCγ2 variant had no protective effect on the risk of progressive supranuclear palsy, amyotrophic lateral sclerosis, Parkinson's disease, or multiple sclerosis (163). On a functional level, the P522R mutation shows an increased enzymatic activity in cell lines compared with WT enzyme, acting as a functional hypermorph (164).

Human genetic data were further corroborated by using well-studied animal models of AD (165, 166). Comparative gene expression profiling of known genetic risk factors in AD revealed an increase of *PLCG2* gene expression in the cortex of A*pp*^NL-G-F/NL-G-F^ mice (165). Single microglia sequencing of two different mouse models of AD, one displaying either Aβ or tau pathology, further confirmed the expression of AD risk genes, including *PLCG2* gene, and also confirmed that more microglia adopt an activated phenotype when facing Aβ than tau pathology (166).

Although PLCγ2 is active in various cell types and tissues as highlighted in this article, within the brain, PLCγ2 is, interestingly, primarily expressed in microglia, in both humans and mice (164) (Fig. 4). A recent study using genetically engineered human induced pluripotent stem cell–derived microglia-like cells found that PLCγ2 activity in microglia regulates multiple processes downstream of TREM2 including survival, myelin phagocytosis, processing of neuronal debris, and metabolism of lipids (Fig. 4), as well as inflammation downstream of TLRs (167). Interestingly, in the absence of TREM2, TLR-dependent PLCγ2 signaling was intensified as a result of aberrant lipid metabolism, resulting in a hyperinflammatory state (167).

Taken together, these results provide further evidence that PLCγ2 may play an important role in AD pathophysiology. Future studies investigating how these variants in PLCγ2 modulate its function and microglia phenotypes in AD could lead to the identification of novel therapeutic strategies.

## Conclusion

In summary, PLCγ2 is a fascinating enzyme regulating diverse biological functions through critical immunological molecules used by a variety of extracellular receptor signaling pathways. Conclusive genetic evidence demonstrates how important normal PLCγ2 function is to immune health, the overaction of which can lead to immunodeficiency, autoimmunity, or autoinflammation. Some of these same mutations can also drive hematopoietic cell proliferation, for example, in Ibrutinib-refractory CLL, and so inhibition of PLCγ2 could be considered as a therapeutic modality in some malignancies and immune disorders. However, such intervention would need to be approached with caution, as the constitutive roles of PLCγ2 are important, as are other gain-of-function variants that provide protection against neurodegenerative disease. Therefore, improved understanding of PLCγ2 functions could inform cellular and temporal targeting of this pathway for therapeutic benefit in the future.

## Conflict of interest

S. L. M. is a scientific advisor for IFM Therapeutics. J. T. J., E. M., and S. L. N. declare no conflicts of interest.
